# Comparative *In Vitro* Study on Magnetic Iron Oxide Nanoparticles for MRI Tracking of Adipose Tissue-Derived Progenitor Cells

**DOI:** 10.1371/journal.pone.0108055

**Published:** 2014-09-22

**Authors:** Annika Kasten, Cordula Grüttner, Jens-Peter Kühn, Rainer Bader, Juliane Pasold, Bernhard Frerich

**Affiliations:** 1 Department of Oral and Maxillofacial Surgery, Facial Plastic Surgery, Rostock University Medical Center, Rostock, Germany; 2 Micromod Partikeltechnologie GmbH, Rostock, Germany; 3 Department of Radiology and Neuroradiology, Greifswald University Medical Center, Greifswald, Germany; 4 Department of Orthopaedics, Biomechanics and Implant Technology Research Laboratory, Rostock University Medical Center, Rostock, Germany; Brandeis University, United States of America

## Abstract

Magnetic resonance imaging (MRI) using measurement of the transverse relaxation time (R2*) is to be considered as a promising approach for cell tracking experiments to evaluate the fate of transplanted progenitor cells and develop successful cell therapies for tissue engineering. While the relationship between core composition of nanoparticles and their MRI properties is well studied, little is known about possible effects on progenitor cells. This *in vitro* study aims at comparing two magnetic iron oxide nanoparticle types, single vs. multi-core nanoparticles, regarding their physico-chemical characteristics, effects on cellular behavior of adipose tissue-derived stem cells (ASC) like differentiation and proliferation as well as their detection and quantification by means of MRI. Quantification of both nanoparticle types revealed a linear correlation between labeling concentration and R2* values. However, according to core composition, different levels of labeling concentrations were needed to achieve comparable R2* values. Cell viability was not altered for all labeling concentrations, whereas the proliferation rate increased with increasing labeling concentrations. Likewise, deposition of lipid droplets as well as matrix calcification revealed to be highly dose-dependent particularly regarding multi-core nanoparticle-labeled cells. Synthesis of cartilage matrix proteins and mRNA expression of collagen type II was also highly dependent on nanoparticle labeling. In general, the differentiation potential was decreased with increasing labeling concentrations. This *in vitro* study provides the proof of principle for further *in vivo* tracking experiments of progenitor cells using nanoparticles with different core compositions but also provides striking evidence that combined testing of biological and MRI properties is advisable as improved MRI properties of multi-core nanoparticles may result in altered cell functions.

## Introduction

Engineering of adipose tissue using adipose tissue-derived progenitor cells has been advocated for the cure of soft tissue defects or for persistent soft tissue augmentation. Different strategies have been proposed, including implantation of suited scaffolds seeded with mesenchymal stem cells, injection of stem cells or progenitor cells using different kinds of carriers like hyaluronic acid gels or particulate carriers [1]. The success of these different engineering strategies depends on various parameters, like the efficacy of cell transplantation, the survival of transplanted cells *in vivo*, the mode of application and the vascularization of the recipient site. In order to monitor the fate *in vivo* and to draw conclusions for developing successful cell therapies, the tracking of the transplanted cells might be helpful and advisable. Magnetic resonance imaging (MRI) has emerged as an excellent method for *in vivo* cell tracking using magnetic nanoparticles because of its high spatial resolution, non-invasiveness and no deposition of ionizing energy [2]–[4].

The applied nanoparticle types differ in core composition resulting in higher iron oxide levels per nanoparticle of multi-core nanoparticles (BNF starch) compared to single core nanoparticles (nanomag-D-spio). It is known that the decay of MR signal is proportional to the iron concentration [5]. This susceptibility effect caused by iron might be useful for cell tracking using iron oxide containing nanoparticles. Different kinds of nanoparticles are commercially available which the manufactures recommend for cell labeling purposes. Especially, superparamagnetic iron oxide nanoparticles (SPIO) are preferentially used for MRI applications due to their properties as they do not retain magnetism after removal of the magnetic field [3]. However, before SPIO nanoparticles are used for cell labeling it is important to know the influence of their physico-chemical properties (e.g. core composition) on the susceptibility effect in MR imaging. In addition, an efficient and rapid internalization of nanoparticles is needed in order to guarantee sufficient labeling of cells for imaging procedures. Magnetic iron oxide particles exhibit highly negatively charged and hydrophobic surfaces leading to aggregation and formation of large clusters diminishing the potential for cellular uptake. To prevent this, nanoparticles are coated with stabilizers like carbohydrates as natural polymers which are added at the time of preparation resulting in a characteristic core-shell architecture [3], [4], [6]. However, carbohydrates on nanoparticle surfaces do not mediate sufficient cellular uptake and, therefore, for instance poly-L-lysine (PLL) known for promoting cell adhesion is applied [2], [7]–[10].

In this study, adipose tissue-derived stem cells (ASC) as multipotent progenitor cells within the adipose tissue were used. The maintenance of their key properties, differentiation into osteogenic, adipogenic and chondrogenic lineages as well as self-renewal, due to nanoparticle labeling is of high importance. But cellular migration and proliferation capacity is known to be affected by high intracellular concentrations of iron oxide nanoparticles [11]–[13]. It was also reported that SPIO-labeled cells exhibit a decreased ability for chondrogenic differentiation *in vitro* whereas adipogenesis and osteogenesis remained unaffected [14], [15]. Overall, particle sizes and dose-dependent effects have to be considered.

The present *in vitro* study aims at comparing two iron oxide containing nanoparticles differing in core composition, BNF starch and nanomag-D-spio, both with a diameter of 100 nm and conjugated with poly-D-lysine (PDL) regarding their potential for stem cell tracking experiments using MRI. Beside their potential use for hyperthermia applications in cancer therapy [16], it is questioned whether both nanoparticle types are adequate for future *in vivo* MRI applications in terms of possible altered stem cell properties and MRI properties. Furthermore, dose-dependent effects will be addressed by using different concentrations of each nanoparticle type for labeling. Both nanoparticle types were characterized by physico-chemical parameters like hydrodynamic diameter and zeta potential. Furthermore, the influence of nanoparticles on cellular functions of adipose tissue-derived was tested particularly with regard to their differentiation potential and proliferation as well as potential cytotoxic effects. Finally, nanoparticle-labeled cells were analyzed in experiments using a high-field 7.1 T animal MRI device.

## Materials and Methods

### Synthesis of nanoparticles

Two types of iron oxide magnetic nanoparticle formulations – bionized nanoferrite (BNF) starch particles and nanomag-D-spio (obtained from micromod Partikeltechnologie GmbH, Rostock-Warnemünde, Germany) were used. Nanomag-D-spio particles were synthesized by alkaline precipitation of iron(II) and iron(III) salts in presence of dextran according to a modified Molday method [17]. Thus, the single iron oxide crystals are surrounded by the dextran matrix to form so-called single core particles. BNF starch particles were prepared by a core-shell method with the alkaline precipitation of iron oxide under high pressure homogenization conditions in the first step, followed by a coating with starch in the second step [18]. This method leads to multi-crystalline core particles with a core of multiple magnetite crystals and a starch shell. For fluorescence imaging and flow cytometric measurements, the dextran surface of the particles was functionalized with amino groups for covalent binding of the red fluorescent (redF) dye DY-555–N-hydroxysuccinimide ester (Dyomics, Germany) to both particle types (nanomag-CLD-redF/BNF starch-redF). For stem cell incorporation, nanomag-D-spio and BNF starch particles or their corresponding fluorescent variants were treated with a solution of poly-D-lysine (PDL; Sigma-Aldrich Chemie GmbH, Munich, Germany) in phosphate buffered saline (PBS; Sigma-Aldrich Chemie GmbH) (pH = 7.4), directly before addition to the cells. The suspension of PDL coated magnetic nanoparticles had final concentrations of 1.5 mg Fe/ml and 15 µg PDL/ml [19].

### Characterization of nanoparticles

The hydrodynamic particle diameter was measured by dynamic light scattering with a Zetasizer Nano-ZS90 (Malvern Instr. Ltd, Malvern, United Kingdom) at a constant iron concentration of 0.2 mg/ml. The zeta potential of the particles was determined by Laser Doppler Electrophoresis also with a Zetasizer Nano-ZS90 (Malvern Instr. Ltd) at a constant iron concentration of 0.2 mg/ml in 1 mM KCl solution at pH = 7.

### Ethics statement

This study was approved by the Ethics Committee of the Rostock University Medical Center. A written consent was signed by every patient prior to surgery.

### Cell culture

Adipose tissue-derived stem cells were isolated from samples of human adipose tissue obtained from the iliac region during surgery of patients (both male and female ranging between 44–76 years of age) in the Department of Oral and Maxillofacial Surgery, Facial Plastic Surgery, Rostock University Medical Center. The adipose tissue was minced with sterile scissors and digested with collagenase (Serva Electrophoresis GmbH, Heidelberg, Germany) at 37°C on a rocking platform (VWR International GmbH, Darmstadt, Germany). The suspension was filtrated using a 100 µm cell strainer (BD Biosciences, San Jose, CA, USA) to remove tissue remnants, centrifuged (300×g, 10 min, room temperature (RT)), and plated in 25 cm^2^ tissue culture flasks (Greiner Bio One, Frickenhausen, Germany). Cells were cultured with culture medium consisting of equal volumes of Iscove's modified Dulbecco's medium and Ham's F12 Nutrient Mix (both from Life Technologies GmbH, Darmstadt, Germany) supplemented with 10% newborn calf serum (v/v; PAA Laboratories, Pasching, Germany), 10 ng/ml human fibroblast growth factor-basic (EMD Millipore Corporation, Billerica, MA, USA) and 1% penicillin-streptomycin (v/v; Life Technologies GmbH) at 37°C in a humified atmosphere with 5% CO2. Differentiation potential of isolated cells was reported previously [20].

### Particle loading of cells

Adherent stem cells were incubated with unlabeled and redF-labeled nanoparticles as follows: BNF starch particles at a concentration of 10, 25, and 50 µg Fe/ml as well as nanomag-D-spio particles at a concentration of 25, 50, and 100 µg Fe/ml. Nanoparticles were diluted in culture medium.

### Verification of nanoparticle internalization

To verify internalization of unlabeled nanoparticles, cells were stained with Prussian Blue and counterstained with nuclear fast red. Briefly, cells were fixed with 4% paraformaldehyde (PFA; AppliChem, Darmstadt, Germany) for 10 min at room temperature (RT) and washed twice with phosphate buffered saline (PBS; Biochrom, Berlin, Germany). Cells were incubated for 20 min in a mixture of 1% HCl (Carl Roth, Karlsruhe, Germany) and 2% potassium ferrocyanide (AppliChem) at equal volumes, washed twice with PBS and counterstained using 1% nuclear fast red (VWR International GmbH) in 5% aqueous aluminium sulfate (AppliChem) solution for 20 min. Cells were washed twice with PBS and once with distilled water. Finally, cells were embedded in mounting medium consisting of 12% poly(vinyl alcohol) (w/v; Sigma-Aldrich Chemie GmbH), 30% glycerol anhydrous (w/v; AppliChem), 0.53 mM phenol (Sigma-Aldrich Chemie GmbH) and 60 mM TRIS (pH 8.5; AppliChem). After polymerization of mounting medium, stained cells were investigated using the microscope Zeiss Axiovert 40 CFL (Carl Zeiss Microscopy GmbH, Jena, Germany).

### Intracellular uptake of nanoparticles

The cellular iron uptake was quantified using a ferrozine assay as described elsewhere [21]. Briefly, cells were seeded with a density of 4×10^5^ cells per cm^2^ and loaded with particles as described above. Following incubation of 24 hours, cells were washed with PBS, detached using 0.5% trypsin/0.2% EDTA (PAA Laboratories), and counted using a hemocytometer. Cells were lyzed using 50 mM NaOH (Carl Roth, Karlsruhe, Germany) and incubated for 2 h at RT on a shaking device. Then, 2 volumes of freshly prepared iron releasing reagent containing 3 parts of 0.5 M HCl and 1 part of 4.5% KMnO_4_ (w/v; VWR International GmbH) was added to cell lysates and incubated for 2 h at 60°C. After cooling down to room temperature, samples were mixed with 0.07 volumes of iron detection reagent containing 6.5 mM FerroZine Iron Reagent (Sigma-Aldrich Chemie GmbH), 6.5 mM neocuproine (Sigma-Aldrich Chemie GmbH), 2.5 M ammonium acetate (EMD Millipore Corporation), and 1 M ascorbic acid (VWR International GmbH) and incubated for 30 min at RT on a shaking device to allow color development. Afterwards, samples were transferred in wells of a 96 microtiter well plate (Greiner Bio One) and absorbance was measured at 550 nm using the bottom-read plate reader Infinite M200 (Tecan Group Ltd., Männedorf, Switzerland). To determine iron concentrations, standard curves of BNF starch particles (0–7.5 µg iron) and nanomag-D-spio particles (0–2.4 µg iron) were prepared as reference. Iron concentrations were divided by cell numbers to calculate iron uptake per cell. The particle uptake was computed by using the number of particles per mg iron.

### Viability/Cytotoxicity assay

To distinguish between live and dead cells, the LIVE/DEAD Viability/Cytotoxicity Kit for mammalian cells (Life Technologies GmbH) was performed according to the manufacturer's instructions. At first, cell nuclei were counterstained with 2 µg/ml cell-permeant nucleic acid stain bisBenzimide H 33342 trihydrochloride (Hoechst 33342; Sigma-Aldrich Chemie GmbH) for 30 min at 37°C. In the following, cells were incubated with 2 nM calcein AM and 4 nM ethidium homodimer-1 for 30 min at 37°C. After washing twice with PBS, cells were examined using the inverted microscope Axio Observer (Carl Zeiss Microscopy GmbH).

### Activity of mitochondrial dehydrogenases

Initially, 1.5×10^4^ cells/cm^2^ were seeded into wells of 24 well plates (Greiner Bio One) and loaded 24 h later with BNF starch particles (10/25/50 µg Fe/ml) as well as nanomag-D-spio particles (25/50/100 µg Fe/ml). After 7 days, the activity of mitochondrial dehydrogenases was determined by conversion of the yellow tetrazolium salt XTT to an orange formazan dye using the Cell Proliferation Kit II (Roche Diagnostics Deutschland GmbH, Mannheim, Germany). The kit was used according to the manufacturer's instructions. Briefly, cells were incubated with 500 µl XTT solution diluted in culture medium for 1.5 h at 37°C. Then, 100 µl of supernatant was transferred into a new 96 microtiter well plate to measure absorbance at wavelength of 600 nm using a microplate reader (Anthos Mikrosysteme GmbH, Krefeld, Germany). To normalize the activity of mitochondrial dehydrogenases to relative cell number, cells were stained with DNA binding crystal violet. Cells were washed twice with PBS, fixed with isopropanol (Carl Roth) for 10 min at RT and washed three times with 0.05% tween-20 (v/v; Carl Roth). Then, 0.1% crystal violet (Sigma-Aldrich Chemie GmbH) was added to the cells and incubated for 15 min at RT on a shaking device. Cells were extensively washed with distilled water until all unbound crystal violet was removed. Bound crystal violet was resolved by adding 33% acetic acid (Avantor Performance Materials, Deventer, Netherlands) for 15 min at RT on a shaking device. In a final step, crystal violet solution was transferred in wells of 96 microtiter well plate to measure absorbance at wavelength of 600 nm using a microplate reader.

### Proliferation

To test proliferation behavior of cells dependent on particle loading, 10^4^ cells/cm^2^ were seeded into wells of 24 well plates. After 24 h, adherent cells were loaded with BNF starch particles (10/25/50 µg Fe/ml) and nanomag-D-spio particles (25/50/100 µg Fe/ml). Proliferation of cells was tested 2, 4, 7, and 10 days after particle loading and investigated using CyQuant Direct Cell Proliferation Assay Kit (Life Technologies GmbH) according to the manufacturer's instructions. Briefly, cells were incubated with culture medium containing CyQuant Direct nucleic acid stain (diluted 1∶500) and CyQuant Direct background suppressor I (diluted 1∶100) for 60 minutes at 37°C. Fluorescence of samples was read using a bottom-read plate reader Infinite M200 (excitation wavelength λ = 480 nm/emission wavelength λ = 535 nm). For constructing a standard curve to convert sample fluorescence values into cell numbers, cells (ranging from 7.5 to 17.5×10^3^ cells/cm^2^) were seeded 6 hours before starting proliferation assay to allow cells to adhere. Proliferation was determined for four individual donors in triplicates.

### Differentiation assays

For *adipogenic differentiation*, 3×10^4^ cells/cm^2^ were seeded and incubated for 21 d with culture medium containing 0.5 mM 3-isobutyl-1-methylxanthine, 10 µM insulin, 1 µM dexamethasone, 8 mg/l biotin, 2.5 mg/l DL-panthothenic acid hemicalcium salt (all from Sigma-Aldrich Chemie GmbH). Adipogenic differentiation was verified by staining of lipid droplets using Bodipy 493/503 (Life Technologies). Cell nuclei were stained with Hoechst 33342 as described above to enable normalization of accumulation of lipid droplets to cell number. Afterwards, cells were washed twice with PBS and fixed with 4% PFA for 30 min at RT. After washing cells three times with PBS, cells were incubated with 250 mg/ml Bodipy 493/503 for 10 min at RT in the dark. Stained cells were then washed twice with PBS and three times with distilled water.

For *osteogenic differentiation*, ASC were seeded with a density of 10^4^ cells/cm^2^ and stimulated for 28 d with an osteogenic differentiation medium consisting of culture medium supplemented with 10 mM β-glycerophosphate disodium salt hydrate, 50 µg/ml L-ascorbic acid 2-phosphate sesquimagnesium salt hydrate, 0.1 µM dexamethasone, and 10 nM 1α-25-dihydroxyvitamin D3 (all from Sigma-Aldrich Chemie GmbH). Osteogenic differentiation was verified by determining matrix calcification of stimulated cells using 5 µg/ml of calcium sensitive fluorescent dye calcein (Sigma-Aldrich Chemie GmbH) overnight at 37°C. Cell nuclei were stained with Hoechst 33342 as described above to normalize matrix calcification to cell number.

Fluorescence of Bodipy 493/503 and calcein as well as cell nuclei was measured using the Infinite M200.

For *chondrogenic differentiation*, ASC were seeded with a density of 10^5^ cells/96 well and incubated for an hour at 37°C under continuous shaking (60 rpm/min) to form pellets. Cell pellets were then cultivated for 21 days at 37°C and 5% CO_2_ in Dulbecco's Modified Eagle Medium (Life Technologies GmbH) containing 50 µg/ml ascorbic acid, 100 nM dexamethasone (both from Sigma-Aldrich Chemie GmbH), 1% ITS (Becton-Dickinson, Heidelberg, Germany), 50 ng/ml IGF-1 (R&D Systems, Wiesbaden, Germany), and 50 ng/ml TGF-ß1 (Tebu-Bio, Offenbach, Germany). Chondrogenic differentiation was verified by determining matrix proteins (collagen types I and II). Therefore, pellets were fixed with 4% PFA for 24 hours, embedded in paraffin, and cut into 4 µm sections which were stained with Heidenhain's AZAN trichrome stain. RNA was isolated with NucleoSpin RNA (MACHEREY-NAGEL, Düren, Germany) according to the manufacturer's instructions and the collagen type II expression was analyzed with Real-Time quantitative PCR (qTower 2.0 and qPCR software 1.0, Analytik Jena, Jena, Germany). Results were normalized to expression of β-actin.

### Localization of nanoparticles by confocal laser scanning microscopy

Nuclei of cells were stained by default using 2 µg/ml Hoechst 33342 for 30 min at 37°C. Staining of mitochondria was performed by using the MitoTracker Green FM (Life Technologies GmbH) according to the manufacturer's instructions. Briefly, cells were washed twice with Hanks' Balanced Salt Solution (Life Technologies GmbH) and incubated with 100 nM MitoTracker solution for 30 min in the dark. Staining solution was removed and culture medium was added.

For lysosome investigations, cells were washed twice with PBS and fixed with 4% PFA followed by permeabiliziation with 0.1% triton X-100 (Sigma-Aldrich Chemie GmbH) both for 10 min at RT. After washing twice with PBS, cells were incubated with a primary antibody against lysosomal-associated membrane protein-1 (LAMP-1) (New England Biolabs, Ipswich, MA, USA) for 6 hours in a 1∶100 dilution. As a secondary antibody, Alexa Fluor 488 goat anti-rabbit IgG (Life Technologies GmbH) was used (diluted 1∶100) and cells were incubated for 30 min at RT in the dark. Cells were washed again and embedded with a cover slip in mounting medium. Investigations of stained cells were performed on a confocal laser scanning microscope LSM 780 (Carl Zeiss Microscopy GmbH, Jena, Germany).

### Flow cytometry assays

Evaluation of fluorescence intensity of nanoparticle-labeled cells was carried out using the BD FACSCalibur Flow Cytometer (BD Biosciences). A total of 50,000 events were acquired per sample. Data were analyzed using FlowJo software version 5.7.2 (Tree Star, Ashland, OR, USA). ASC were labeled with 50 µg Fe/ml BNF starch-redF and 100 µg Fe/ml nanomag-CLD-redF nanoparticles and cultured for one day unless otherwise stated. If cells were confluent, they were splitted 1∶3 to allow appropriate cell proliferation. Afterwards, cells were trypsinized, washed with PBS, and fixed in BD CellFix (BD Biosciences). Unlabeled cells served as control.

### *In vitro* magnetic resonance imaging of cell phantoms

Cells were seeded at a density of 6.5×10^5^ in 25 cm^2^ tissue culture flask and labeled with nanoparticles as described before. After 24 h, cells were detached, fixed with 4% PFA, washed twice with PBS, and counted using a hemocytometer. For preparing the cell phantoms, 6.3×10^5^ cells were embedded into 15 ml 1.5% agarose (Carl Roth) followed by application of ultra sound to remove air bubbles.

MRI of cell phantoms was performed twice using a high-field 7.1 Tesla animal MRI system (ClinScan, Bruker Corp., Billerica, MA, USA). A gradient-echo sequence was acquired using the following sequence parameters: TR: 33 ms; TE_1_/TE_2_/TE_3_/TE_4_/TE_5_/TE_6_: 2.0/3.5/4.4/5.4/6.3/7.3 ms; flip angle: 3°; matrix: 128×128 interpolated; field of view: 42 mm; 100%; averages: 1, echo train length: 1; slice thickness: 1.5 mm; 16 slices. Signal decay of all four echo times were assessed and a R2* maps (R2* = 1/T2*) were calculated using Matlab (Mathworks, Natik, MA, USA) [22]. Known confounders for R2* mapping such as noise bias and multi-spectral complexity of fat were corrected whereas R2* maps were reconstructed as previously described [23]. One observer (JK) realized the image analysis using the freeware Osirix (Pixmeo, Bernex, Switzerland). For each cell concentration, respectively iron concentration, a rectangle region of interest (ROI) based measurement of the R2* was performed. ROIs were placed over the whole container of each iron concentration. Care was taken to exclude artifacts, which are typically present in the boundary of the containers.

### Statistics

Experiments were repeated at least four times using ASC of individual donors to ensure reproducibility. Statistical analyses were performed on using IBM SPSS Statistics version 20.0.0 (IBM Corp., Armonk, NY, USA). To test normal data distribution, the Kolmogorov-Smirnov test was used. In case of normal distributed data, data were evaluated using Tukey *post-hoc* test following one-way analysis of variance (ANOVA). If data were not normally distributed, the Kruskal-Wallis one-way analysis of variance was used followed by Mann-Whitney U-test as *post-hoc* test. Significant differences were marked at three levels (*p<0.05, **p<0.01, ***p<0.001). All graphs were created using SigmaPlot 12.5 software (Systat Software, Inc., San Jose, CA, USA). According to data distribution, graphs display mean ± standard deviation (SD) or box-and-whisker diagrams unless otherwise stated. Boxes include 25^th^ and 75^th^ percentiles as well as the median. Whiskers represent 10^th^ and 90^th^ percentiles. To test linear correlation between two variables, Pearson correlation coefficient (R^2^) was calculated using SigmaPlot 12.5. A correlation coefficient of 1 demonstrates a perfect correlation.

## Results

### Characterization of nanoparticles

BNF starch nanoparticles as multi-crystalline core particles and nanomag-D-spio nanoparticles as single core particles were characterized by hydrodynamic diameter (HD), polydispersity index (PI), and zeta potential in plain, PDL-coated, redF-plain and redF-PDL-coated variants at a constant iron concentration of 0.2 mg/ml, respectively. Results of nanoparticle characterization are demonstrated in Table 1. Dynamic light scattering of BNF starch plain/redF plain and nanomag-D-spio plain/redF-plain nanoparticles indicated average HD of 119 nm/148 nm and 86 nm/130 nm, respectively, revealing smaller HD for the single core particles. In general, the hydrodynamic diameter of plain nanoparticles was significantly increased due to the surface functionalization with amino groups followed by covalent binding of the fluorescent dye. Furthermore, PDL coating of plain and redF-plain variants of both nanoparticle types resulted in an increased HD. The PI of all nanoparticle variants ranged between 0.11 and 0.23 demonstrating monodisperse size distributions. Analysis of the zeta potential data revealed clearly negative values for BNF starch plain and nanomag-D-spio plain nanoparticles of −18±1.4 mV and −14±1.3 mV, respectively. The PDL coating shifted the zeta potential of the plain particles by 10–13 mV into positive direction to improve the surface charge of the particles for cell uptake. The introduction of amino groups and the conjugation of the fluorescent dye led to a significant positive shift of the zeta potential of fluorescent nanoparticles. The PDL coating did not further change the zeta potential of fluorescent BNF starch nanoparticles and stabilized the positive zeta potential of the nanomag-CLD-spio nanoparticles.

**Table 1:** Physico-chemical characterization of nanoparticles.

	Hydrodynamic diameter (nm)	Polydispersity index	Zeta potential (mV) mean ± SD
**BNF starch**			
plain	119	0.11	−18±1.4
PDL	140	0.21	−5±1.1
redF plain	148	0.12	−1±1.1
redF PDL	185	0.2	1±1.0
**nanomag-D-spio**			
plain	86	0.18	−14±1.3
PDL	93	0.22	−4±1.0
**nanomag-CLD-redF**			
plain	130	0.23	12±1.3
PDL	167	0.23	6±1.1

Both nanoparticle types including their redF-labeled and PDL-coated variants were characterized by determination of hydrodynamic diameter, polydispersity index, and zeta potential.

### Dose- and type-dependent cell labeling

**Figure 1:**
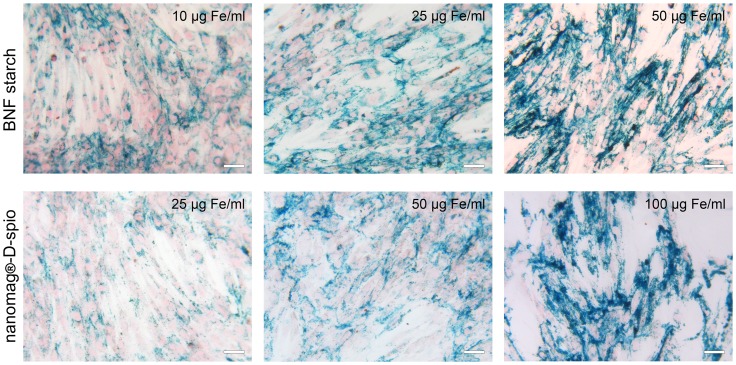
Nanoparticle internalization verified by Prussian Blue staining. After incubation of ASC with BNF starch nanoparticles (10/25/50 µg Fe/ml) and nanomag-D-spio nanoparticles (25/50/100 µg Fe/ml), iron oxide of internalized particles was visualized by Prussian Blue staining (Zeiss Axiovert 40 CFL, Carl Zeiss Microscopy GmbH, Jena, Germany; scale bars  = 50 µm).

To verify whether ASCs were labeled with BNF starch particles or nanomag-D-spio particles, the Prussian Blue staining was performed to detect the iron within the cells following nanoparticle incubation. The reduction of ferric to ferrous iron results in a formation of blue precipitates and indicates the presence of nanoparticles. The results clearly indicate the presence of both nanoparticle types within the cells (Fig. 1). Moreover, higher nanoparticle labeling concentrations led to an increased Prussian Blue staining indicating a dose-dependent labeling process independent of the nanoparticle type. To assess cell labeling efficiency, representative flow cytometry distributions of fluorescence intensity in a cell population exposed to red fluorescent BNF starch-redF and nanomag-CLD-redF nanoparticles are given in Figure S1 revealing single-peaked mean fluorescence intensity signals of nanoparticle-labeled cells. Furthermore, mean fluorescence intensities of labeled cells revealed 5-fold higher values for BNF starch-redF-labeled cells (21.5±4.6) and nanomag-CLD-redF-labeled cells (19.6±2.5) compared to unlabeled cells (3.6±0.6).

For quantifying the uptake of nanoparticles in dependence of particle type and dose, an adapted ferrozine assay [21] was performed. BNF starch-labeled cells revealed a number of particles per cell (mean ± SD) of 1933±1478, 6823±2980 and 21278±10019 at labeling concentrations of 10 µg Fe/ml, 25 µg Fe/ml and 50 µg Fe/ml, respectively (Table 2). Regarding nanomag-D-spio-labeled cells, 6162±1475, 16420±3119 and 38736±3008 nanoparticles per cell (mean ± SD) were internalized if labeling concentrations of 25 µg Fe/ml, 50 µg Fe/ml and 100 µg Fe/ml were used, respectively. Consistently with Prussian Blue staining, a dose-dependent nanoparticle uptake was detected resulting in a higher nanoparticle uptake with increasing nanoparticle labeling concentrations. No significant differences were found for the number of nanoparticles per cell at labeling concentrations of 25 µg Fe/ml and 50 µg Fe/ml between BNF starch and nanomag-D-spio-labeled cells.

**Table 2:** Nanoparticle uptake of labeled ASC.

	BNF starch			nanomag-D-spio		
	min	max	mean ± SD	min	Max	mean ± SD
10 µg Fe/ml	58	5171	1933±1478	-	-	-
25 µg Fe/ml	1519	15469	6823±2980^a^	2259	9234	6162±1475^a^
50 µg Fe/ml	9095	41045	21278±10019^b^	9836	22688	16420±3119^b^
100 µg Fe/ml	-	-	-	32375	45266	38736±3008

Uptake of nanoparticles was determined using a ferrozin assay and displayed as number of particles per cell. Shown is the maximal and minimal as well as mean ± SD number of particles per cell.

a,b no significant difference between cellular uptake of BNF starch and nanomag-D-spio nanoparticles (Mann-Whitney U-test).

### Cytotoxicity studies

**Figure 2:**
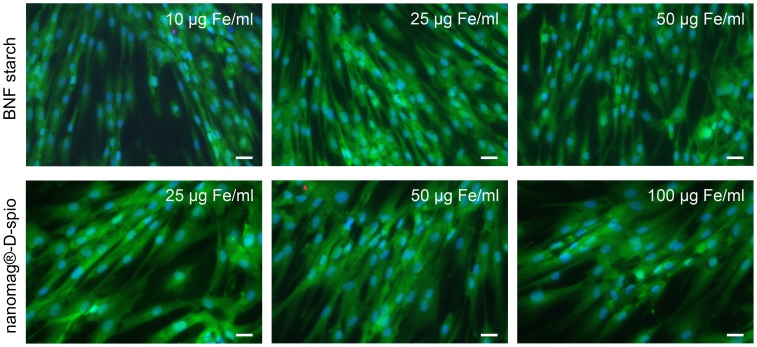
Viability/Cytotoxicity test after nanoparticle labeling. After treatment of ASC with BNF starch nanoparticles (10/25/50 µg Fe/ml) and nanomag-D-spio nanoparticles, a viability/cytotoxicity assay was performed up to 14 days following labeling. Viable cells and nuclei of apoptotic cells were stained with calcein AM (green) and ethidium homodimer (red), respectively. Cells were counterstained with Hoechst 33342 (blue). No cytotoxic effects were detected due to nanoparticle labeling (Axio Observer, Carl Zeiss Microscopy GmbH, Jena, Germany; scale bars  = 50 µm).

Potential cytotoxicity of nanoparticles was analyzed using a viability/cytotoxicity assay, staining viable cells green and apoptotic cells red. Up to 14 days, the viability of nanoparticle-labeled cells was monitored and results are exemplarily shown for day 7 in Fig. 2. Cell labeling with BNF starch or nanomag-D-spio particles did not affect cell viability. These results indicate that there is no evidence for cytotoxic effects of BNF starch particles and nanomag-D-spio particles at concentrations up to 50 µg Fe/ml and 100 µg Fe/ml, respectively.

**Figure 3:**
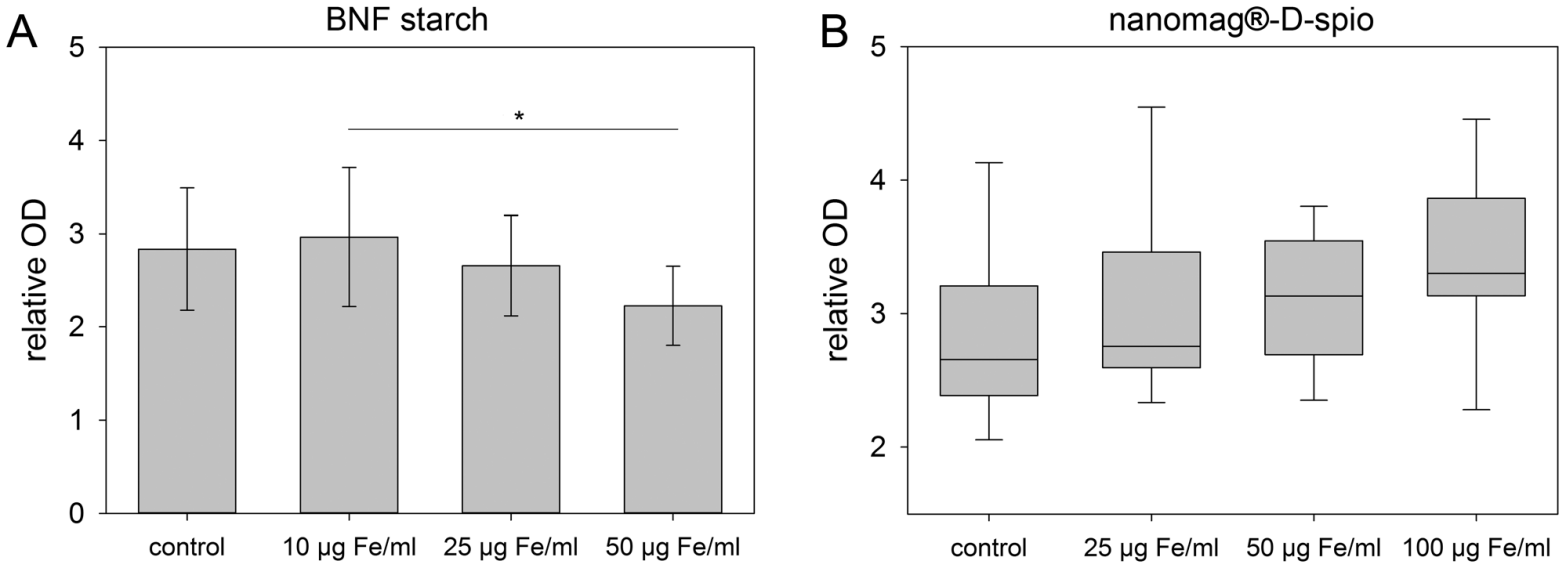
Activity of mitochondrial dehydrogenases. Cells were treated with (A) BNF starch nanoparticles and (B) nanomag-D-spio nanoparticles. The activity of mitochondrial dehydrogenases was determined at day 7 after labeling. Only BNF starch labeling of ASC at the highest concentration of 50 µg Fe/ml diminished the activity of mitochondrial dehydrogenases significantly compared to control cells (n = 4; (A) mean ± SD, ANOVA/Tukey's-test; (B) boxplot, Mann-Whitney U-test; *P<0.05, **P<0.01, ***P<0.001).

In order to investigate the biocompatibility of nanoparticles, the activity of mitochondrial dehydrogenases as a parameter for mitochondrial function was determined and no significant effects of BNF starch and nanomag-D-spio particles on ASC compared to control cells were found (Fig. 3).

### Proliferation of nanoparticle-labeled ASC

**Figure 4:**
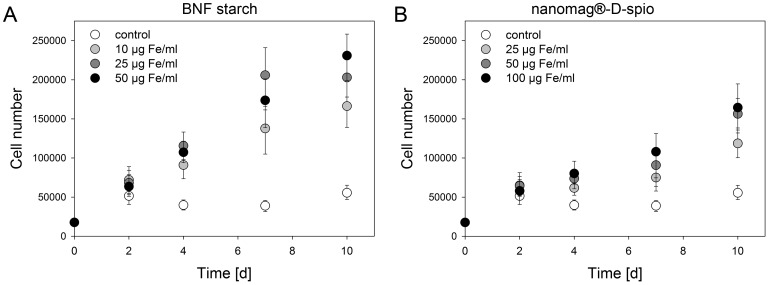
Proliferation of nanoparticle-labeled ASC. Cells were treated with (A) BNF starch nanoparticles and (B) nanomag-D-spio nanoparticles and cell numbers were determined up to 10 days after labeling. Cell treatment with both nanoparticle types resulted generally in a higher proliferation rate compared to control cells (n = 4; median, error bars represent 25^th^ and 75^th^ percentiles).

The analysis of cell proliferation revealed an increased proliferation in dependence of nanoparticle labeling (Fig. 4) whereupon the degree of increase was highly dependent on the nanoparticle type. Cell labeling using BNF starch particles resulted in a higher proliferation rate compared to nanomag-D-spio loaded cells. The comparison of total cell numbers between BNF starch and nanomag-D-spio particles revealed on day 10 a comparable cell number if the lowest BNF starch particle concentration (10 µg Fe/ml) and the highest nanomag-D-spio particle concentration (100 µg Fe/ml) were used. Starting on day 4, the differences between cell numbers were significant for each labeling concentration independent of the nanoparticle type. To determine the effect of cell proliferation on nanoparticle distribution within the BNF starch-redF-labeled cell population, the fluorescence intensity of cells was analyzed by flow cytometry (Figure S2). Two days after the addition of nanoparticles, the cell population of labeled cells could be clearly distinguished from the cell population of unlabeled cells. After 14 days, however, labeled cells showed a broad distribution of fluorescence intensity ranging between control cells and two days labeled cells.

### Impact of nanoparticle labeling on ASC differentiation

**Figure 5:**
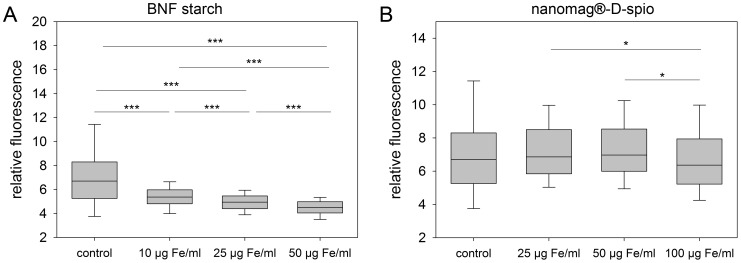
Effect of labeling on adipogenic differentiation of ASC. After cell labeling with (A) BNF starch nanoparticles and (B) nanomag-D-spio nanoparticles, adipogenic differentiation conditions were provided for 21 days and lipid droplet deposition was measured. BNF starch labeling of ASC resulted in a dose-dependent reduction of adipogenic differentiation potential (n = 4; boxplots, Mann-Whitney U-test; *P<0.05, **P<0.01, ***P<0.001).

The differentiation of ASC into mature adipocytes was characterized by deposition of lipid droplets which was measured 21 days after adipogenic induction in dependence of nanoparticle labeling (Fig. 5). In comparison to control cells, nanomag-D-spio-labeled cells demonstrated a comparable adipogenic differentiation potential whereupon labeling with BNF starch particles resulted in a concentration-dependent reduction in adipogenic differentiation. Even BNF starch particles at 10 µg Fe/ml were sufficient to decrease lipid droplet deposition.

**Figure 6:**
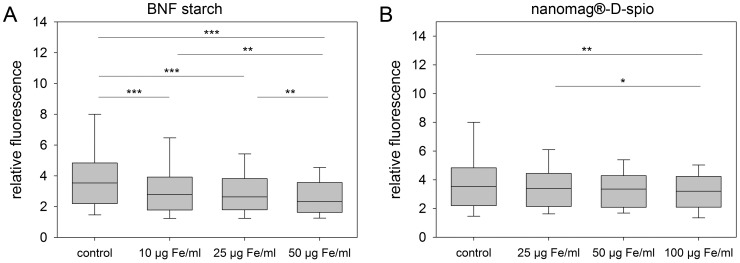
Effect of labeling on osteogenic differentiation of ASC. Cells were labeled with (A) BNF starch nanoparticles and (B) nanomag-D-spio nanoparticles. Osteogenic differentiation conditions were provided for 28 days following measurement of matrix calcification. BNF starch labeling of ASC resulted in a dose-dependent reduction of osteogenic differentiation potential whereas only nanomag-D-spio labeling at the highest concentration (100 µg Fe/ml) had a diminishing effect on the matrix calcification (n = 5; boxplots, Mann-Whitney U-test; *P<0.05, **P<0.01, ***P<0.001).

The osteogenic differentiation of ASC into osteoblasts is accompanied by extracellular matrix calcification. Therefore, matrix calcification of particle-labeled ASC was analyzed to evidence ostogenic differentiation (Fig. 6). Whereas application of nanomag-D-spio particles did not affect adipogenic differentiation potential, osteogenic differentiation was decreased at the highest particle concentration of 100 µg Fe/ml compared to control cells. Due to cell labeling with BNF starch particles, the osteogenic differentiation showed a similar concentration-dependent decrease like the adipogenic differentiation. However, no significant differences between particle labeling at 10 µg Fe/ml and 25 µg Fe/ml were detected. In general, the application of the highest nanoparticle concentration, independently of the particle type, resulted in the lowest matrix calcification.

**Figure 7:**
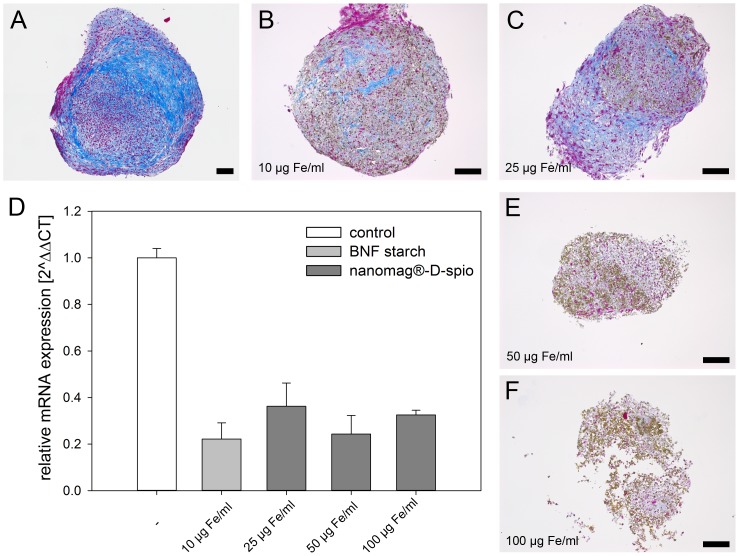
Effect of labeling on chondrogenic differentiation of ASC. (A) Unlabeled, (B) BNF starch- and (C, E, F) nanomag-D-spio-labeled cells were chondrogenically stimulated for 21 d. Pellets were analyzed using Heidenhain's AZAN trichrome staining showing a reduced collagen type II-positive extracellular matrix (light blue) due to nanoparticle labeling. BNF-labeled cells (25 and 50 µg Fe/ml) failed to generate compact pellets (not shown) (Axio Imager M2, Carl Zeiss Microscopy GmbH, Jena, Germany; scale bars  = 100 µm). (D) Collagen type II was analyzed using Real-Time quantitative PCR revealing a diminished mRNA expression of collagen type II due to nanoparticle labeling (2∧ΔΔCT ± %CV; n = 2, normalized to β-actin).

The chondrogenic differentiation of unlabeled and nanoparticle-labeled cells in pellet cultures was characterized by Heidenhain's AZAN trichrome staining of collagen type I (dark blue) and II (light blue) as well as mRNA expression of collagen type II (Fig. 7). In general, pellets of nanoparticle-labeled cells were not as dense as pellets of unlabeled cells, especially if high concentrations of nanomag-D-spio (100 µg Fe/ml) were used. Labeling with high BNF starch concentrations (25 and 50 µg Fe/ml) completely prevented the formation of pellets. AZAN trichrome staining revealed highly positive areas for cartilage matrix proteins, in particular collagen type II, in pellets of unlabeled cells. Positive staining was diminished due to nanoparticle labeling in a dose-dependent manner. The same could be shown for mRNA expression of collagen type II which was decreased approximately twofold for nanoparticle-labeled cells compared to unlabeled cells.

### Localization of nanoparticles

**Figure 8:**
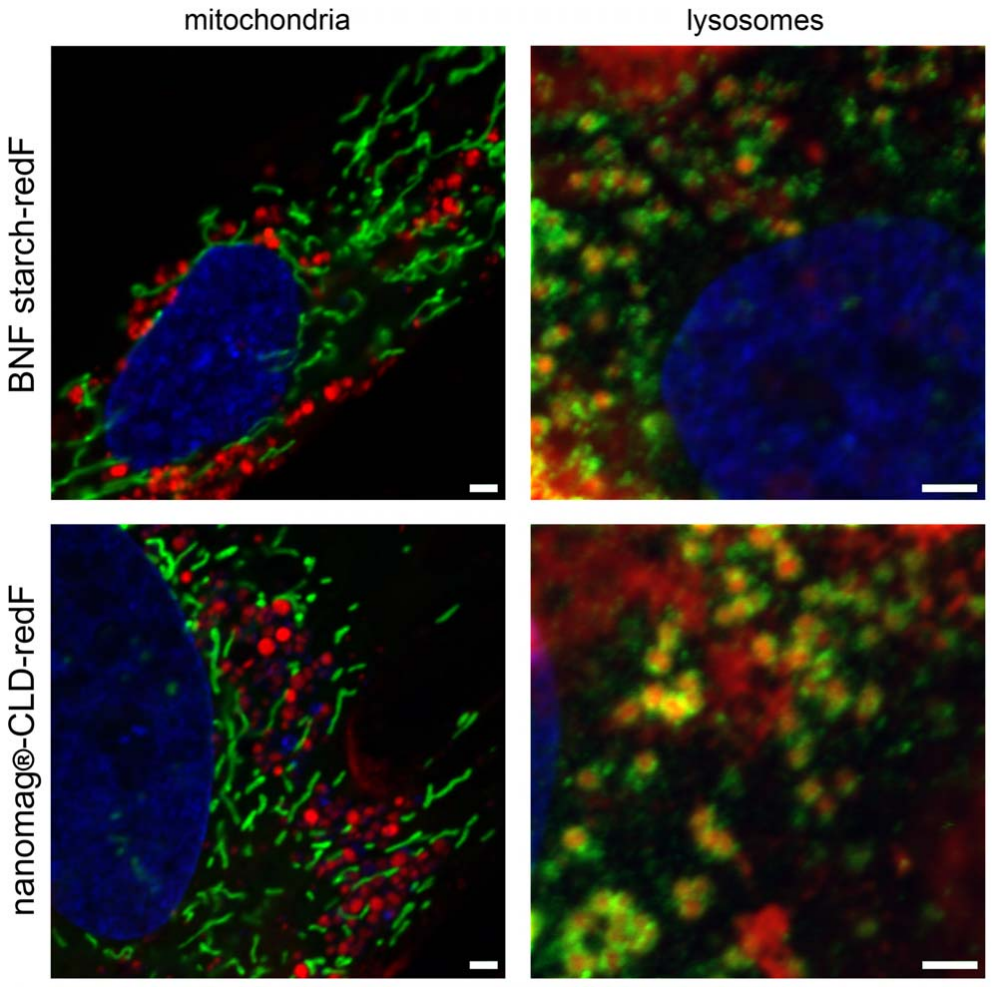
Intracellular localization of nanoparticles. Confocal laser scanning images of ASC labeled with red fluorescent variants of nanoparticles, BNF starch-redF (25 µg Fe/ml) or nanomag-CLD-redF (50 µg Fe/ml), and stained 24 h after labeling for mitochondria and lysosomes (LAMP-1) (both shown in green). Cell's nuclei were counterstained with Hoechst 33342 (blue). No co-localization between nanoparticles and mitochondria or nuclei was found. Both nanoparticle types appear to be associated with lysosomes 24 hours after labeling. In each case, overlay images are shown (LSM 780, Carl Zeiss Microscopy GmbH, Jena, Germany; scale bars  = 2 µm).

On confocal laser scanning microscopy images, both the internalized BNF starch and nanomag-D-spio particles coated with DY555 (red) appeared to localize to punctate structures distributed throughout the cytoplasm both 6 h and 24 h after labeling (Fig. 8). To determine which cellular compartment these structures possibly represent, cells were stained for mitochondria as well as endosomal and lysosomal compartments. Additionally, cells were counterstained by using nuclei stain Hoechst 33342 (blue) whereupon co-localization was not found. Mitochondria were visualized using a live-cell imaging assay. Up to 24 hours after cell labeling, no co-localization between both nanoparticles types and mitochondria was observed. Due to the fact that the lysosomal metabolism of superparamagnetic iron oxide nanoparticles has been demonstrated [24], labeled cells were stained with an antibody against the lysosomal-associated membrane protein-1 (LAMP-1). An association between the nanoparticles and the lysosome was detected 24 h after nanoparticle-labeling.

### Visualization of nanoparticle-labeled cells using MRI

**Figure 9:**
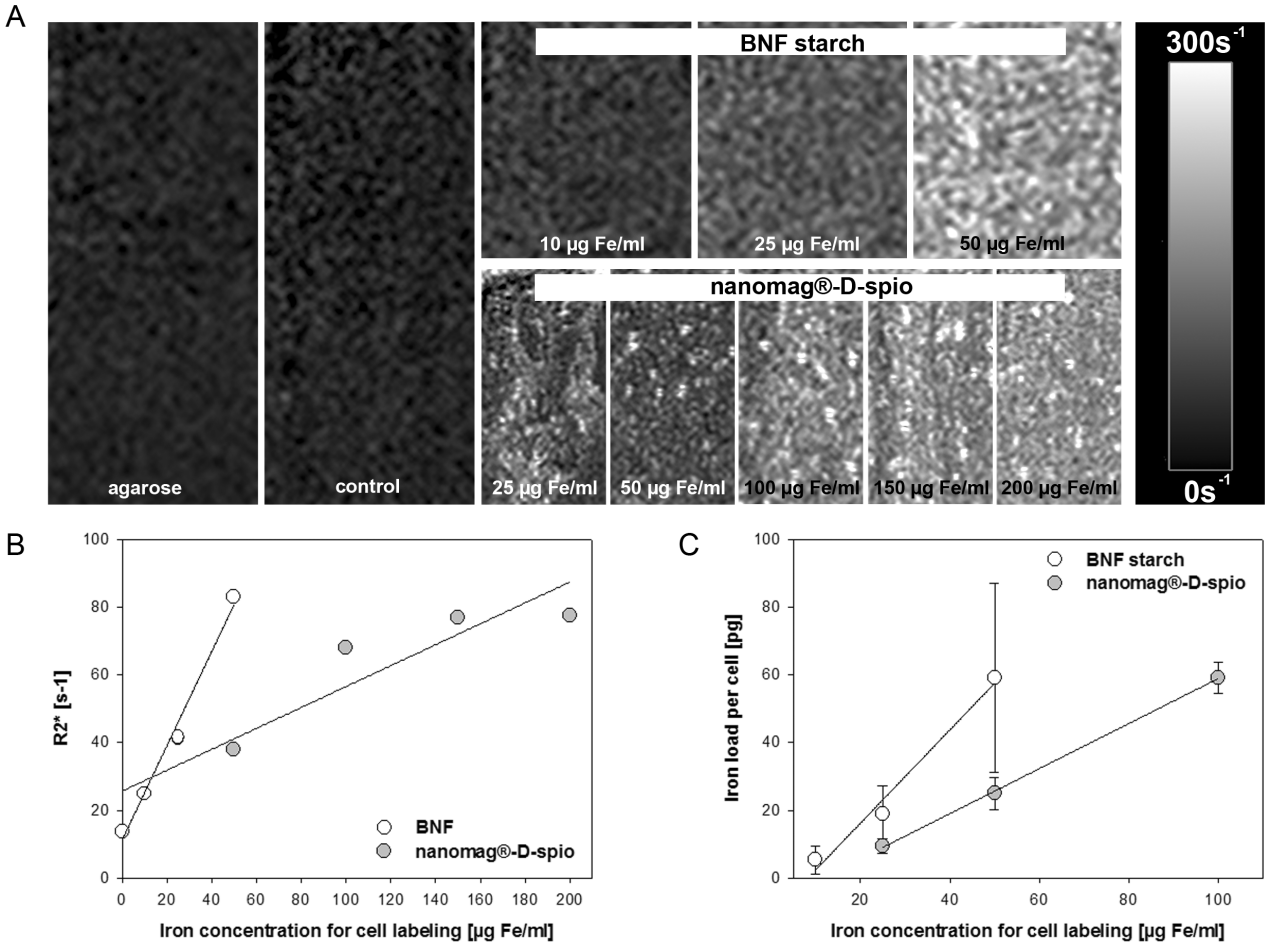
Correlation between iron content per cell and R2* values. R2* maps of cell phantoms are shown in (A). The (B) R2* values of cell phantoms as well as (C) iron load per cell are shown as a function of applied iron concentration for cell labeling. BNF starch and nanomag-D-spio labeled ASC were labeled with 0–50 µg Fe/ml and 0–200 µg Fe/ml, respectively. For cell phantoms, cells were embedded in 1.5% agarose and MRI was performed using a high-field 7.1 Tesla animal MR system (Clin Scan, Bruker Corp., Billerica, MA, USA). Pure agarose without cells served also as control. Iron load per cell was quantified using a ferrozine assay (n = 5, mean ± SD). For both nanoparticle types, the iron load per cell correlated with R2* values as a function of the applied iron concentration for cell labeling.

R2* maps of cell phantoms are shown in Fig. 9A. For both nanoparticle types, R2* was increased as a function of the iron concentration used for ASC labeling. However, the images clearly show a homogeneous distribution of BNF starch compared to an inhomogeneous claggy distribution of nanomag-D-spio. Fig. 9B presents the relationship between R2* times and iron concentration of both nanoparticle types used for ASC labeling showing an excellent linear correlation for BNF starch-labeled ASC (R^2^ = 0.989); respectively for nanomag-D-spio-labeled ASC (R^2^ = 0.856). However, there are different slopes of R2* and both nanoparticle types, e.g. the slope of R2* and BNF starch-labeled ASC is much stronger compared to nanomag-D-spio-labeled ASC. According to nanoparticle composition, a higher level of labeling concentrations of nanomag-D-spio was needed to achieve BNF starch comparable R2* values. This fact was confirmed by the measurement of iron concentration per cell of both nanoparticle types (Fig. 9C).

## Discussion

Magnetic nanoparticles are widely used in *in vivo* applications like hyperthermia, as contrast agents, in drug delivery systems or for cell tracking experiments [4], [25]–[29]. For assessing the therapeutic efficacy of cell transplantations, it is increasingly important to track implanted cells e.g. via magnetic resonance imaging [4]. This *in vitro* study is aiming at comparing two iron oxide containing nanoparticle types of different composition regarding their (i) physico-chemical characteristics, (ii) influence on cellular parameters of ASC like differentiation or proliferation, and (iii) MRI properties. Overall, this study represents the initial step to establish a convenient *in vivo* tracking model combining ASC, magnetic iron oxide nanoparticles and MRI for adipose tissue engineering strategies.

Despite the similarities of both nanoparticle types in iron oxide composition, they exhibit substantial differences in intercrystal structure, polymer coating, and crystal size, the latter demonstrated by transmission electron microscopy of iron oxide crystals [16]. Carbohydrates like dextran or starch are known to mediate only insufficient cellular uptake of nanoparticles [10], [14]. Therefore, BNF starch and nanomag-D-spio particles were coated with poly-D-lysine (PDL) to mediate non-specific internalization [3]. To achieve an equivalent loading of ASC with both nanoparticle types at equal labeling concentrations, charge and size of nanoparticles have to be comparable as both properties influence particle uptake [30]–[32]. Confirming this, uptake of BNF starch PDL and nanomag-D-spio PDL nanoparticles was comparable at labeling concentrations of 25 µg Fe/ml and 50 µg Fe/ml correlating with similar zeta potentials of both nanoparticle types. However, differences in hydrodynamic diameter of BNF starch PDL and nanomag-D-spio PDL nanoparticles showed no effect on nanoparticle uptake. Internalization of both nanoparticle types was determined using Prussian Blue staining confirming a dose-dependent cell labeling. The uptake of nanoparticles per cell was analyzed by an adapted ferrozin assay [21]. Likewise the Prussian Blue staining, a dose-dependent effect of labeling was detected. Moreover, no significant differences in nanoparticle uptake at labeling concentrations of 25 µg Fe/ml and 50 µg Fe/ml were found between both nanoparticle types indicating that surface modification with PDL was equally effective to ensure particle internalization. Furthermore, distinct distribution with a 5-fold higher mean fluorescence intensity as well as single-peaked mean fluorescence intensity signals of nanoparticle-labeled cells compared to unlabeled cells indicate that total cell population was labeled and no cell population subsets with lower degrees of nanoparticle uptake exist. Thus, PDL-coating of nanoparticles seems to be highly efficient in terms of nanoparticle uptake.

Cytotoxicity can potentially be induced through nanoscale properties of nanoparticles influencing major cell components like mitochondria and nuclei. Therefore, cell exposure to SPION with high exposure levels is associated with significant toxic effects like chromosome condensation, formation of apoptotic bodies, and impaired mitochondrial function [33]. In this study, cytotoxic effects were tested by checking the plasma membrane integrity and intracellular esterase activity as well as the activity of mitochondrial dehydrogenases (MDH). Overall, no significant cytotoxic effects of both nanoparticle types on ASC were detected. Both nanoparticle types did not directly interact with mitochondria or nuclei because co-localization was not detected. Regarding the internalization pathway, an association of both nanoparticle types with lysosomes (24 hours after labeling) was confirmed. Results are consistent with previous studies reporting that PDL induces non-specific particle internalization resulting in the incorporation of nanoparticles into endosomes and trafficking to lysosomes. Afterwards, the acidic environment of lysosomes promotes the degradation of iron oxide nanoparticles releasing free iron into the cytoplasm [4], [6], [24], [33].

Contradictory reports exist how the application of iron oxide containing nanoparticles influences the proliferative behavior of cells [2], [12], [30], [34], [35]. In this study, an increase in cell proliferation was observed independently of the nanoparticle type and in a concentration-dependent manner. These results are consistent with Huang et al. [34], who traced this phenomenon back to a diminished intracellular H_2_O_2_ level and an altered cell cycle protein regulator profile. Nevertheless, a direct comparison between the two nanoparticle types revealed a higher proliferative capacity for BNF starch particles. As the uptake of BNF starch and nanomag-D-spio particles is comparable at labeling concentrations of 25 µg Fe/ml and 50 µg Fe/ml and, generally, BNF starch particles contain a higher amount of iron oxide, the degradation profile of both nanoparticle types may differ resulting in varying amounts of iron released from lysosomes into the cytoplasm and becoming available for metabolic pathways [4], [24], [36], [37]. Increasing intracellular iron levels stimulate the synthesis of ferritin, a major iron storage protein, which acts as a mitogen to mediate cell proliferation [38]–[40]. The effect of cell proliferation on nanoparticle labeling was demonstrated in an exemplary manner for BNF starch-redF-labeled cells. The broad distribution of fluorescence intensity of nanoparticle-labeled cells 14 days after labeling indicates that cell population subsets with varying degrees of nanoparticle incorporation exist and seems to correlate with the high proliferation rate of BNF starch-labeled cells. Results imply that cell divisions diluted the concentration of nanoparticles per cell as it was reported earlier [41], [42]. Internalized BNF starch-redF nanoparticles were detected up to 14 days within the cells by flow cytometry but at this point the timeline of nanoparticle clearance within the cells remains unclear. Huang et al. still observed nanoparticle signals in cells after a retention time of 3 weeks [43].

Adipose tissue-derived mesenchymal stem cells are capable of multipotent differentiation including adipogenic, osteogenic, and chondrogenic differentiation lineages [44], [45]. According to several studies, the capacity of mesenchymal stem cells to adipogenesis or osteogenesis is not altered due to particle labeling [2], [14], [30], [35], [46], [47]. However, it was revealed in this study that BNF starch particles at all labeling concentrations diminished both adipogenic and osteogenic differentiation in a dose-dependent manner. Regarding nanomag-D-spio particles, only osteogenic differentiation of labeled cells (100 µg Fe/ml) was decreased compared to control cells. A dose-dependent inhibitory effect of SPIO on the osteogenic differentiation was reported by Chen et al. [48] suggesting the involvement of Wnt/β-catenin signaling-mediated MMP2 expression. The activation of Wnt signaling has also been shown to inhibit osteogenic differentiation [49]–[51]. Moreover, increasing iron levels are able to activate the canonical Wnt/β-catenin pathway by regulating β-catenin [52]. However, activation of Wnt/β-catenin signaling is also discussed to promote osteogenic differentiation by regulating e.g. miR-346 and miR-218 [53], [54]. Additionally, this study revealed that typical marker for chondrogenesis like collagen type II were diminished at the RNA- and protein-level due to nanoparticle labeling. Apparently, BNF starch at high labeling concentrations disabled cells completely to generate three-dimensional pellets. These results are in line with several other studies analyzing the effect of iron oxide nanoparticles on chondrogenesis [14], [30], [55]. Whereas it was reported that SPIO labeling interfered with the organization of F-actin resulting in a dose-dependent impairment of chondrogenesis [30], F-actin staining of nanoparticle-labeled cells did not reveal any disorganization of F-actin (data not shown). The mechanism of iron oxide containing nanoparticle labeling on chondrogenesis remains at this point vague.

MRI is a promising approach for cell tracking experiments. R2* mapping as a special MR technique is able to quantify iron concentrations and might allow an indirect estimation of iron concentration in labeled cells. According to a higher concentration of iron oxide in multi-core nanoparticles compared to single core nanoparticles, an increased iron upload per cell was found in BNF starch-labeled cells. Results indicate that iron load per cell and R2* values are similarly correlated with iron concentration for cell labeling suggesting that core composition influences R2* values. As cell division leads to nanoparticle dilution among daughter cells [41], [42], dilution effects have to be considered regarding MRT detection of nanoparticle-labeled cells and may present a limiting factor. So far, proliferation as well as differentiation behavior of ASC *in vivo* remains unknown and will be addressed in the future.

R2* mapping is based on a multi-echo gradient echo sequence. In theory, if in-phase/opposed phase echo times are chosen, the chemical shift encoded MRI approach allows a quantification of iron and also fat. Recent studies demonstrated the feasibility of quantification of tissue fat and iron simultaneous using a multi-echo chemical shift encoded MRI [56]. Further studies will follow to prove the feasibility of *in vivo* cell tracking of BNF starch and nanomag-D-spio nanoparticle-labeled ASC in adipose tissue engineering approaches using a multi-echo chemical shift encoded MRI.

## Conclusions

Our findings provide the proof of principle of our *in vitro* model by demonstrating that both nanoparticle types are feasible for tracking experiments of progenitor cells in Tissue Engineering strategies using a MRI approach and, thereby, offering a new scope of application besides their potential use in hyperthermia treatment for cancer therapy. Further studies will follow to prove the feasibility of *in vivo* cell tracking of BNF starch and nanomag-D-spio nanoparticle-labeled ASC in adipose tissue engineering approaches. As results impressively demonstrate, BNF starch as multi-core nanoparticles exerts greater influence on differentiation and proliferation potential of ASC than single core nanomag-D-spio nanoparticles. Effects remain, nevertheless, dose-dependent. Thus, for *in vivo* experiments the nanoparticle concentration has to be chosen carefully to ensure both integrity and MRI detection of the adipose tissue-derived progenitor cells.

## Supporting Information

Figure S1 **Efficiency of nanoparticle labeling.** ASC were treated with 50 µg Fe/ml BNF starch-redF and 100 µg Fe/ml nanomag-CLD-redF nanoparticles for 24 h. (A) Typical distributions of fluorescence intensity of nanoparticle-labeled cells compared to unlabeled control cells as measured by flow cytometry. (B) Mean cell fluorescence intensity was obtained by flow cytometry (mean ± SD, n = 3).
(TIF)

Figure S2 **Effect of proliferation on nanoparticle labeling of ASC.** ASC were labeled with 50 µg Fe/ml BNF starch-redF nanoparticles. Representative distributions of fluorescence intensity are given two days as well as 14 days after labeling compared to unlabeled control cells using flow cytometry.
(TIF)
