# Supplementary figures and images for: Comparative In Vitro Study on Magnetic Iron Oxide Nanoparticles for MRI Tracking of Adipose Tissue-Derived Progenitor Cells
Source: PLoS One. 2014 Sep 22;9(9):e108055. doi: 10.1371/journal.pone.0108055 (PMC4171509; doi:10.1371/journal.pone.0108055)

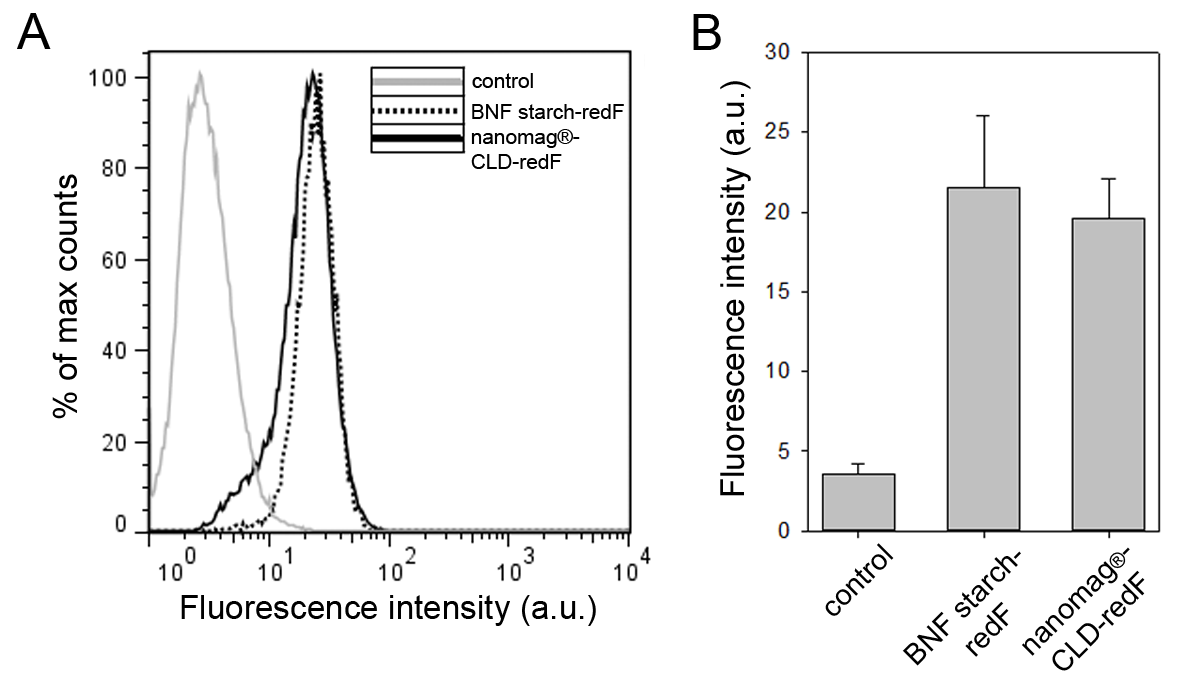

Supplement: Figure S1 — Efficiency of nanoparticle labeling. ASC were treated with 50 µg Fe/ml BNF starch-redF and 100 µg Fe/ml nanomag-CLD-redF nanoparticles for 24 h. (A) Typical distributions of fluorescence intensity of nanoparticle-labeled cells compared to unlabeled control cells as measured by flow cytometry. (B) Mean cell fluorescence intensity was obtained by flow cytometry (mean ± SD, n = 3). (TIF) [file pone.0108055.s001.tif]

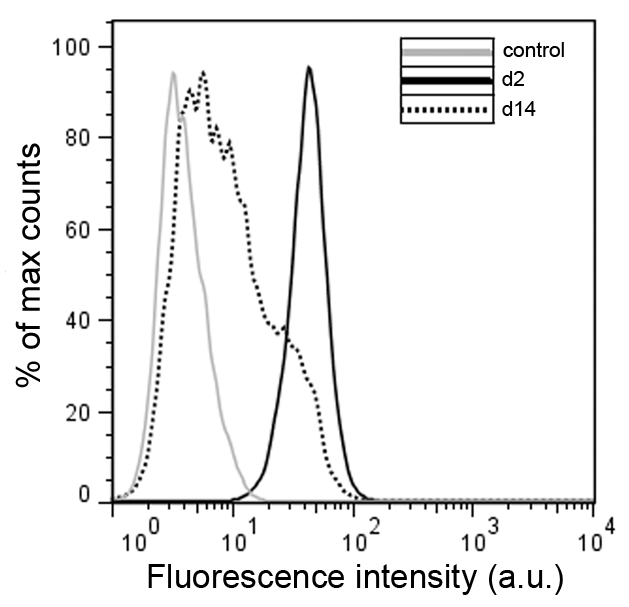

Supplement: Figure S2 — Effect of proliferation on nanoparticle labeling of ASC. ASC were labeled with 50 µg Fe/ml BNF starch-redF nanoparticles. Representative distributions of fluorescence intensity are given two days as well as 14 days after labeling compared to unlabeled control cells using flow cytometry. (TIF) [file pone.0108055.s002.tif]
